# Where we live matters: a comparison of chronic pain treatment between remote and non-remote regions of Quebec, Canada

**DOI:** 10.3389/fpain.2024.1291101

**Published:** 2024-02-26

**Authors:** Claudie Audet, Meriem Zerriouh, Hermine Lore Nguena Nguefack, Nancy Julien, M. Gabrielle Pagé, Line Guénette, Lucie Blais, Anaïs Lacasse

**Affiliations:** ^1^ Département des Sciences de la Santé, Université du Québec en Abitibi-Témiscamingue (UQAT), Rouyn-Noranda, QC, Canada; ^2^ Department of Anesthesiology and Pain Medicine, Centre de Recherche Centre Hospitalier de l’Université de Montréal (CRCHUM), Montréal, QC, Canada; ^3^ Département d’Anesthésiologie et de Médecine de la Douleur, Faculté de Médecine, Université de Montréal, Montréal, QC, Canada; ^4^ Faculté de Pharmacie, Université Laval, Québec, QC, Canada; ^5^ Population Health and Optimal Health Practices Axis, Centre de Recherche CHU de Québec—Université Laval, Québec, QC, Canada; ^6^ Faculté de Pharmacie, Université de Montréal, Montréal, QC, Canada

**Keywords:** chronic pain, remote region, treatment, access, rural, multimodal, pharmacological, non-pharmacological

## Abstract

**Objective:**

Where a person lives is a recognized socioeconomic determinant of health and influences healthcare access. This study aimed to compare the pain treatment profile of persons with chronic pain (CP) living in remote regions to those living in non-remote regions (near or in major urban centers).

**Methods:**

A cross-sectional study was performed among persons living with CP across Quebec. In a web-based questionnaire, participants were asked to report in which of the 17 administrative regions they were living (six considered “remote”). Pain treatment profile was drawn up using seven variables: use of prescribed pain medications, over-the-counter pain medications, non-pharmacological pain treatments, multimodal approach, access to a trusted healthcare professional for pain management, excessive polypharmacy (≥10 medications), and use of cannabis for pain.

**Results:**

1,399 participants completed the questionnaire (women: 83.4%, mean age: 50 years, living in remote regions: 23.8%). As compared to persons living in remote regions, those living in non-remote regions were more likely to report using prescribed pain medications (83.8% vs. 67.4%), a multimodal approach (81.5% vs. 75.5%), experience excessive polypharmacy (28.1% vs. 19.1%), and report using cannabis for pain (33.1% vs. 20.7%) (bivariable *p* < 0.05). Only the use of prescribed medications as well as cannabis remained significantly associated with the region of residence in the multivariable models.

**Discussion:**

There are differences in treatment profiles of persons with CP depending on the region they live. Our results highlight the importance of considering remoteness, and not only rurality, when it comes to better understanding the determinants of pain management.

## Introduction

Quebec is a Canadian province with a population of 8,604,495 (1) spread over a territory of 1,700,000 km^2^ (2). Within this territory, there are six geographical areas qualified as remote resource regions which are described as regions geographically distant from major urban centers such as Montreal and Quebec City (1). These six regions shelter 10% of the Quebec population (1). In remote and sparsely populated regions around the world, the organization, planning and delivery of healthcare are challenging due to a number of factors such as finances, logistic, and human resources (3). These challenges can lead to difficulties for locals to access care, something that urban dwellers experience to a lesser extent (4, 5), and even compromise the equity of access to healthcare (6). Thomas and Perchansky (7) listed a number of important aspects of healthcare accessibility such as availability, accommodation, affordability, and patient acceptability. In terms of availability and accessibility, it appears that only 8% of the physicians practising in Canada are serving the 18% of the Canadian population who are living in remote regions (8). Moreover, health services are few and scattered and inhabitants of remote regions often have to travel long distances to access the services they need (9, 10), making care access even more difficult. In a context where remote populations are generally older, less wealthy, and sicker (4, 9, 11), it is conceivable that the region of residence and care access are major social determinants of health (12, 13).

Although remote and rural regions can be associated with similar healthcare access issues (6) and they are often named interchangeably with mistake (14), they differ as remote regions are characterized by their distance from major population hubs (14) as opposed to rural regions which are defined as areas where population density is lower than an urban setting (15). Remote regions seem far less studied in the health literature (6 times fewer hits on the PubMed electronic database in June 2023). Studies conducted in the United States (16), Australia (17), and Canada (18) show disparities in the health status of persons living in remote regions. For instance, higher prevalence of chronic diseases (16) and overweight persons (18) and higher mortality rates (17) have been reported for residents of remote regions compare to those from non-remote regions. In terms of chronic pain, various Canadian, American, and international studies also report a higher prevalence in populations living in rural or remote regions (19–23). For example, while the worldwide prevalence of chronic pain is 20% (24), approximately 27% of the rural American population report living with chronic pain (22). Moreover, Bath et al. (20) reported that Canadians living in rural or remote regions were 30% more likely to have chronic low back pain compared to Canadians living in urban regions.

In terms of treatment, it is largely accepted that chronic pain should be managed using a multimodal approach combining pharmacological, physical, and psychological components (25–27). However, access to multidisciplinary pain clinics as well as to non-pharmacological pain treatments and medical specialists is sometimes more difficult in remote regions (28, 29). Canadian data is scarce regarding whether patients from remote regions present the same treatment profile for chronic pain as patients from major urban centers. Thus, this study aimed to compare the pain treatment profiles of persons with chronic pain living in remote regions to the profiles of those living in non-remote regions (near or in major urban centers). Specifically, this study focused on seven outcomes: the use of prescribed pain medications, over-the-counter pain medications, non-pharmacological pain treatments, multimodal approach, in addition to access to a trusted healthcare professional for pain management, excessive polypharmacy, and cannabis use for pain management. Evidence and consideration of these specificities are important to help decision makers adapt the healthcare organization and the services offered in remote regions.

## Materials and methods

### Study design and data source

This cross-sectional study was conducted using data from the ChrOnic Pain treatment (COPE) Cohort (30), a self-reported data infrastructure resulting from a web-based recruitment of 1,935 French-speaking adults living with chronic pain [persistent or recurrent pain for more than three months (31)] across the province of Quebec (Canada). Between June and October 2019, participants were invited to complete a web-based questionnaire that included validated composite scales and all the indicators identified as a minimum data set by the Canadian Registry Working Group of the Strategy for Patient-Oriented Research Chronic Pain Network (32). The implementation and analysis of the COPE Cohort received ethics approval from the Université du Québec en Abitibi-Témiscamingue's research ethics committee (#2018-05, Lacasse, A.). Informed consent for participation was completed online by participants. For further information, the complete methodology of the COPE Cohort implementation is described in Lacasse et al. (30).

Online self-reported data collection allowed the research team to reach participants throughout the Quebec Territory, with all six remote regions represented. In our sample, 24% of the participants lived in a remote region of Quebec, while the population in remote regions across Canada is around 12% (33). The COPE Cohort, participants' characteristics (pain characteristics, age, level of education, employment status) have been found to be similar to random samples of adults living with chronic pain, both in Canada and around the world (30). Women are, however, overrepresented [84% vs. 55%–65% in Canadian chronic pain random samples (30)], justifying multivariable analyses in order to limit the risk of bias. The COPE Cohort is nevertheless diversified and includes more than 220 men, allowing the assessment of valid multivariable associations.

### Selection criteria

The present study used self-reported data from the sample of participants who completed the region of residence section of the web questionnaire (*n* = 1,399/1,935, 72.3%). No significant differences were found between the study sample and the excluded participants (*p* > 0.05) in terms of age (average of 49.97 years vs. 44.31 years), proportion of women (83.66% vs. 85.71%), average pain intensity (5.4/10 vs. 5.5/10), pain duration and pain location.

### Study variables

The questionnaire included items on pain location, circumstances surrounding its onset, duration, frequency, intensity, neuropathic component, interference, physical function, anxiety and depressive symptoms, age, gender identity, and employment status. Items were also selected following the core outcome domains and measures suggested by the Initiative on Methods, Measurement and Pain Assessment in Clinical Trials (34, 35), the Canadian minimum data set for chronic low back pain research (36), and variables assessed in the Quebec Pain Registry (37).

#### Region of residence

The questionnaire included one item where participants had to indicate the geographical region of Quebec where they currently live (the 17 Quebec administrative regions were listed, along with an additional “other” option which allowed classifying participants who chose to report their city instead of their region). Six of the seventeen regions of the province are qualified as remote resource regions by the Quebec government (1). We therefore separated participants into two comparison groups for analysis, those living in one of the six remote regions and those living in non-remote regions.

#### Pain treatment profile

The pain treatment profile was drawn up using seven dichotomous variables: (1) The use of prescribed pain medications [*“For the treatment of your pain, are you currently using prescribed medications (that require a prescription from a doctor, pharmacist, or nurse practitioner)?”* yes/no]; (2) The use of over-the-counter pain medications [*“For the treatment of your pain, are you currently using over-the-counter medications (that do not require a prescription)?”* yes/no; examples were provided]; (3) The use of non-pharmacological pain treatments [*“Apart from medications, are you currently using any other types of treatments for your pain?”;* A standardized list of 31 treatment options, inspired by Canadian Agency for Drugs and Technologies in Health (CADTH) (38) and Canadian Pain Task Force (39) reports was presented to participants. Participants who checked at least one of these options were considered as users]; (4) The use of a multimodal approach [At the analysis stage, multimodal approach for pain management was defined as the combination of pharmacological and non-pharmacological pain treatments (25)]; (5) Access to a trusted healthcare professional for pain management [*“Do you have access to a healthcare professional you trust for the treatment of your pain (e.g., doctor, nurse, pharmacist, physiotherapist, psychologist, etc.)?”* yes/no]; (6) Excessive polypharmacy [“*How many different medications do you currently use (whether prescribed or over-the-counter; whether they are for pain or any other health problem)?”;* This number was measured using a pull-down menu and was recategorized into excessive polypharmacy [utilization of ≥10 medications (40)] or not; and (7) cannabis use for pain [*“In the past year, have you used cannabis for pain management?”* yes/no].

#### Covariables

In the present analysis, we considered the following sociodemographic characteristics: age, gender identity, Indigenous identity, country of birth, employment status, and postsecondary education. Chronic pain characteristics included pain location, frequency, duration, average intensity in the last 7 days (0–10 numerical rating scale), neuropathic component according to the DN4 (41). Also, tendency toward pain catastrophizing was measured using a single item from the Pain Catastrophizing Scale (42) referred to as “catastrophizing” in the NIH Minimal Dataset for Chronic Low Back Pain (43) and in the STarT Back Screening Tool (44). Pain interference [Brief Pain Inventory (45)] and general pain relief brought by treatments (0%–100%) were also measured. Health profile and lifestyle variables included general health [12-Item Short Form Survey v2 subscale (46)], physical functioning [12-Item Short Form Survey v2 subscale (46)], and emotional distress [Patient Health Questionnaire-4 (47)]. Participants were also screened for alcohol or drug use [“*In the past year, have you felt you wanted or needed to cut down on your drinking or drug use?*” never/rarely/sometimes/often; item from the Canadian minimum data set for chronic low back pain research (36)], cigarette smoking (never smoked, current smoker, used to smoke, but have now quit), and recreational cannabis use (yes/no).

### Statistical analysis

The characteristics of the study population were summarized using descriptive statistics (frequencies, percentages, means, standard deviations) for persons living in remote regions and persons living in non-remote regions separately. Pain treatment profiles (drawn up with the seven above-mentioned variables) were then compared within these two groups by conducting Chi-square and Fisher's exact tests (crude/bivariable analysis). In order to isolate the potential impact of the region of residence (vs. differences explained by other factors unequally distributed in the two groups), seven multivariable logistic regression models were used to investigate the relationship between each pain treatment profile dependent variable and living in a remote region. Covariables listed above and included in all logistic regression models were chosen *a priori* according to the literature and clinical relevance. The different treatment profile variables were included in the models in which they were not considered as the dependent variable. The Hosmer-Lemeshow test confirmed the goodness of fit for each logistic regression (Supplementary Material S1). Results are presented as adjusted odds ratios (OR) along with their 95% confidence intervals (CI). Analyses were conducted using IBM SPSS Statistics 19®.

## Results

A total of 1,399 participants out of 1,935 in the COPE Cohort (72.3%) answered the question about the region of residence (23.8% reported living in one of the six remote regions of the province). Table 1 presents the characteristics for both participants living in remote regions and participants living in non-remote regions. Participants living in remote regions and those living in non-remote regions were similar in terms of age (mean age: 47.1 vs. 50.9). Also, no clinically important differences were found in terms of gender identity (woman: 83.8% vs. 83.6%), country of birth (Canada: 99.1% vs. 95.1%), Aboriginal identification (3.1% vs. 1.4%), and disability benefits (4.8% vs. 6.8%). However, a higher proportion of persons living in remote regions were employed (52.4% vs. 31.0%) and had a postsecondary education level (81.0% vs. 78.5%). In terms of pain characteristics, a lower proportion of persons living in remote regions reported generalized pain (25.8% vs. 38.4%), multisite pain (84.7% vs. 90.2%) and constant pain (79.5 vs. 89.2%). Average pain intensity in the last 7 days was quite the same for both groups (5.1 vs. 5.5).

**Table 1:** Characteristics of the sample (*n* = 1,399).

** **	Participants living in remote regions (*n* = 333)	Participants living in non-remote regions (*n* = 1,066)
Age (years), *mean ± SD*	47.10 ± 13.08	50.87 ± 13.25
Gender identity, *n* (%)	** **	** **
Women	279 (83.78)	888 (83.62)
Men	52 (15.62)	172 (16.19)
Non-binary	2 (0.60)	2 (0.19)
Aboriginal identity, *n* (%)	10 (3.07)	15 (1.44)
Country of birth, *n* (%)		
Canada	328 (99.09)	1,003 (95.07)
Other	3 (0.91)	52 (4.93)
Employed, *n* (%)	174 (52.41)	327 (31.02)
Disability benefits, *n* (%)	16 (4.83)	71 (6.76)
Postsecondary education, *n* (%)	265 (81.04)	827 (78.46)
Generalized pain, *n* (%)	86 (25.83)	409 (38.37)
Multisite pain (≥2 sites), *n* (%)	282 (84.68)	961 (90.15)
Constant pain (vs. intermittent), *n* (%)	264 (79.52)	946 (89.16)
Pain duration (≥10 years), *n* (%)	144 (43.24)	584 (54.94)
Pain intensity on average in the past 7 days (0–10), *mean ± SD*	5.05 ± 2.03	5.53 ± 1.91
Pain interference Brief Pain Inventory score (0–10), *mean ± SD*	5.01 ± 2.34	5.81 ± 2.08
Neuropathic pain DN4 (yes vs. no), *n* (%)	148 (44.44)	592 (56.69)
Tendency toward pain catastrophizing (yes vs. no), *n* (%)	159 (48.18)	647 (60.81)
Psychological distress (PHQ-4), *n* (%)		
None (score 0–2)	98 (31.41)	234 (22.96)
Mild (score 3–5)	116 (37.18)	347 (34.05)
Moderate (score 6–8)	60 (19.23)	234 (22.96)
Severe (score 9–12)	38 (12.18)	204 (20.02)
Physical functioning (SF-12 subscale), *mean ± SD*	36.31 ± 12.06	30.76 ± 9.66
Perceived general health (SF-12 subscale), *mean ± SD*	39.49 ± 12.73	35.39 ± 12.58

Table 2 shows the results of the crude (bivariable) analyses comparing the pain treatment profile among participants living in remote regions and those living in non-remote regions. Within the seven variables of interest, four presented statistically significant differences (*p* < 0.05) when confounding factors were not accounted for. As compared to persons living in remote regions, those living in non-remote regions were more likely to report using cannabis for pain (20.7% vs. 33.1%, *P *< 0.01), experience excessive polypharmacy (19.1% vs. 28.1%, *P *= 0.01), report using prescribed pain medications (67.4% vs. 83.8%, *P *< 0.01), and have a multimodal pain management approach (75.5% vs. 81.5%, *P *= 0.017). In the crude analysis, no statistically significant differences were found between the two groups in terms of the use of over-the-counter pain medication, the use of non-pharmacological pain treatments, and access to a trusted healthcare professional for pain management.

**Table 2:** Crude (bivariable) comparisons of pain treatment profiles.

** **	Participants living in remote regions (*n* = 333)	Participants living in non-remote regions (*n* = 1,066)	*P* values
Use of prescribed pain medications, *n* (%)	223 (67.37)	890 (83.80)	<0.001
Use of over-the-counter pain medications, *n* (%)	222 (67.07)	710 (68.85)	0.942
Use of non-pharmacological pain treatments, *n* (%)	284 (85.29)	909 (85.27)	0.835
Use of multimodal approach, *n* (%)	249 (75.45)	863 (81.49)	0.017
Access to a trusted healthcare professional for pain management, *n* (%)	258 (77.71)	872 (82.11)	0.074
Excessive polypharmacy (use of ≥10 medications), *n* (%)	60 (19.11)	287 (28.14)	0.001
Use of cannabis for pain management, *n* (%)	65 (20.70)	338 (33.07)	<0.001

Table 3 presents the results of the adjusted logistic regression models conducted to investigate the relationships between the region of residence and the pain treatment profile variables. Independently from all other variables included in the models, living in a remote region was only associated with a decreased likelihood of using prescribed pain medications (adjusted OR* *=* *0.6, 95% CI: 0.4–0.9) and using cannabis for pain (adjusted OR* *=* *0.6, 95% CI: 0.4–0.9). Complete results of the multivariable models are presented in Supplementary Material S1.

**Table 3:** Logistic regression models main results.

** **	Living in a remote region (yes vs. no) Adjusted OR^a^ (95% CI)
Use of prescribed pain medications	**0.60** (**0.41–0.87)**
Use of over-the-counter pain medications	1.02 (0.74–1.41)
Use of non-pharmacological pain treatments	0.89 (0.56–1.40)
Use of multimodal approach	0.76 (0.51–1.14**)**
Access to a trusted healthcare professional for pain management	0.82 (0.65–1.20)
Excessive polypharmacy (use of ≥10 medications)	0.94 (0.63–1.40)
Use of cannabis for pain management	**0.60** (**0.38–0.94)**

OR, odds ratio; CI, confidence interval.

Statistically significant associations (*p* < .05) are reported in bold.

^a^ Adjusted for age, gender identity, Aboriginal identity, country of birth, employment, postsecondary education, pain localization, frequency, duration, intensity, neuropathic component, and interference, tendency toward pain catastrophizing, psychological distress, physical functioning, consumption of alcohol or drugs more than intended, perceived general health, cigarette smoking, use of cannabis for other illnesses or recreative purpose. The treatment profile variables were included in the models in which they were not considered as the dependent variable.

## Discussion

To our knowledge, no study had previously portrayed chronic pain treatment in remote vs. non-remote regions of Canada. The present study compared the pain treatment profiles of persons with chronic pain living in remote regions to the profiles of those living in non-remote regions among a large sample of participants from all administrative regions of Quebec (*n* = 1,399). In this study, use of prescribed pain medications, a multimodal approach, excessive polypharmacy and use of cannabis for pain management varied according to the region of residence, but some of these associations are most likely explained by other sociodemographic and clinical factors. In fact, multivariable analysis showed that persons with chronic pain living in remote regions were less likely to use prescribed pain medications as well as cannabis for pain. These results reveal some differences in the pain treatment profiles of patients depending on their region of residence, and underline the importance of considering remoteness as a predictor or confounder when studying pain management. It is important to note that remoteness differs from rurality, since remote regions are characterized by their distance from major population hubs (14). Urban areas (towns and cities) are present in remote regions and rural areas can be close to major urban centers.

### Use of prescribed pain medications

Independently from all other variables included in the model, living in a remote region was associated with a decreased likelihood of using prescribed pain medications. Existing literature on prescribing practices in remote regions seems scarce and unspecific between concepts of rurality and remoteness. Nevertheless, the results of the present study are not entirely consistent with the few available studies on the subject. According to an Australian study, general practitioners are more likely to prescribe opioids if they are practising in regional and remote regions (48). This prescribing trend in remote regions may be influenced by limited access to health services offering non-opioid treatments (49). In Canada, most of the facilities offering multidisciplinary pain treatments are concentrated in large urban centers (50). Patients with chronic pain living in remote regions must therefore refer to their family physician for pain management (50), who may be less inclined to prescribe pain-specific medication due to his or her generalist scope of practice. As our study adjusted for factors such as having access to a trusted healthcare professional for pain management and using non-pharmacological pain treatments, this result should be investigated further in order to understand why persons in remote regions of the province of Quebec use fewer prescribed pain medications than those in major urban centers (e.g., qualitative studies with primary care and pain clinics prescribers in both remote and non-remote regions, quantitative study using health insurance claims on the number of contacts with prescribers). Other contributing factors that could vary from one region to another and are not covered in our study could also be explored, such as economic factors (e.g., income, perceived economic burden of medication use), cultural and societal factors, access to health infrastructure, awareness, and education.

### Use of cannabis for pain management

In our study, persons living in remote regions were less likely to report using cannabis for pain than those living in non-remote regions (21% vs. 33%). Our results are consistent with existing literature comparing cannabis use in remote (or rural) vs. urban regions. An American study investigated the evolution of cigarette and cannabis use from 2007 to 2017 and showed that the prevalence of cannabis use was lower in rural regions (51). Similar findings have been recorded in studies reporting less risky cannabis use in remote regions (52) and a higher prevalence of cannabis and alcohol use in more central and densely populated regions (53). Interestingly, urban car drivers would also have higher odds of testing positive for cannabis than car drivers from remote and rural regions (54). Our findings could be explained by disparities in access to cannabis retail stores between urban and remote regions of Canada where recreational cannabis is legal since 2018. In fact, a recent American study showed that as retailers opened closer to where people live, more individuals used marijuana more frequently (55). Moreover, the 2021 Quebec cannabis survey revealed that distance from cannabis stores is one of the reasons for not buying cannabis in the last 12 months (56). Data collection for the present study was carried out in 2019, just after the legalization of recreational cannabis in 2018. As the first cannabis stores were set up in major urban centers, persons living in remote regions may not have had access to them at the time of data collection; for example, the first cannabis stores of the remote regions of Abitibi-Témiscamingue, Nord-du-Québec and Côte-Nord opened only in 2020 (57–59). In addition, cannabis consumption appears to be on the rise as the number of cannabis stores in the province of Quebec increases and facilitating access to legal cannabis (56). We should keep in mind that the efficacy and safety of cannabis for chronic pain management remains to be demonstrated (60).

### Accounting for confounding factors

The crude analyses conducted in the present study revealed statistically significant differences between persons living in remote regions and those living in non-remote regions in terms of use of prescribed pain medications, use of multimodal approach, excessive polypharmacy as well as use cannabis for pain management. However, following multivariable analyses, only two of these variables remained associated with the region of residence (decreased likelihood of using prescribed pain medications or cannabis for pain management). In the literature, we still encounter underpowered studies that do not apply multivariable analysis when studying factors associated with chronic pain and its treatment (61). Chronic pain and its treatment have many concurrent bio-psycho-social components that have to be taken into account (25, 26, 31). Our results reinforce the importance of applying multivariable analyses when studying real-world treatment of chronic pain. Multivariable analysis allows for the simultaneous analysis of multiple variables and controls for confounding (62). In other words, it is possible to isolate the effects of specific variables of interest and provides a more comprehensive understanding of complex relationships.

### Strengths and limitations

The present study has several strengths such as the utilization of a large community sample of adults living with chronic pain from all regions of Quebec (as opposed to many chronic pain studies conducted among individuals from large urban centers and with contact with the healthcare system). The large sample size enhanced the statistical power and allowed the inclusion of several variables related to pain treatment profiles. There are still some limitations that need to be highlighted. First, the COPE Cohort (30) is a self-reported data infrastructure where participants were self-selected, introducing the possibility of selection as well as information bias (over-representation of women, memory bias, socially desirable responses, under-reporting). However, these biases are most likely non-differential since they affect both comparison groups equally, are not specific to the present study and do not compromise its potential for comparing the pain management profiles of persons living in remote regions and non-remote regions, as the information was collected in the same way in both groups. Second, longitudinal health insurance claims were not analyzed in the present study. Future studies could address this issue by targeting aspects such as access to healthcare and precribers by region of residence. Third, future studies addressing pain treatment in remote regions should include virtual care as a treatment modality, which has developped considerbly since the pandemic. Limited access to the internet for remote vs. non-remote populations should also be considered. Finally, data were cross-sectional, which limits the assessment of temporality between independent and dependent variables and the possibility to reveal causal effects. Future longitudinal studies could be useful to determine longitudinal pain treatment trajectories by region of residence over time. We cannot exclude the possibility that certain covariables included in the multivariable models (e.g., employement) were in the causal pathway between the place of residence and our outcomes of interest. This is why it could be interesting for future studies to explore such relationships using a directed acyclic graphs (DAG) approach.

## Conclusion

Despite its limitations, this study revealed differences in treatment profiles of persons with chronic pain depending on where they live, with persons from remote regions less likely to use prescribed medication and cannabis for pain. On the other hand, the crude (bivariable) differences found in terms of excessive polypharmacy and use of a multimodal pain management approach appear to be explained by other sociodemographic and clinical factors identified in this study. Multivariable differences could be explained by the long distance separating remote regions from urban centers, and the difficulties in accessing healthcare services and cannabis providers. Our results highlight the importance of considering remoteness, not only rurality, when it comes to better understand the determinants of pain management. The unique challenges faced by people living in remote regions make them a distinct entity (63) for whom a one-size-fits-all treatment strategy might not be the most appropriate (63). Pain research must be inclusive, reaching populations currently under-represented in studies (64), such as people living outside major urban centers. In this regard, future studies focusing on pain management should pay particular attention to this issue and include participants from major urban centers, as well as remote and rural regions. Exploring the interaction between remoteness and rurality is also relevant.

## Data Availability

The data analyzed in this study is subject to the following licenses/restrictions: The Cope Cohort dataset is not readily available because participants did not initially provide consent to open data. The data that support the findings of this study are available from the corresponding author upon reasonable request and conditionally to a proper ethical approval for a secondary data analysis. Programming codes can be obtained directly from the corresponding author. Requests to access these datasets should be directed to anais.lacasse@uqat.ca.

## References

[1] Institut de la statistique du Québec. Panorama des régions du Québec (2022).

[2] Gouvernement du Québec. Géographie du territoire québécois (2022). Available online at: https://www.quebec.ca/gouvernement/portrait-quebec/geographie-territoire#c65360 (accessed November 9, 2022).

[3] Young TK Chatwood S Ford J Healey G Jong M Lavoie J Transforming health care in remote communities: report on an international conference. BMC Proc. (2016) 10(6):6. 10.1186/s12919-016-0006-0 28813543 PMC4989899

[4] DesMeules M Pong R Lagacé C Heng D Manuel D Pitblado R How Healthy are Rural Canadians? An Assessment of Their Health Status and Health Determinants. Ottawa, Canada: Canadian Institute for Health Information (2006).

[5] Mikkonen J Raphael D. Social Determinants of Health: The Canadian Facts. Toronto, Canada: York University School of Health Policy and Management (2010).

[6] Bradford NK Caffery LJ Smith AC. Telehealth services in rural and remote Australia: a systematic review of models of care and factors influencing success and sustainability. Rural Remote Health. (2016) 16(4):1–23. 10.22605/RRH4268 27817199

[7] Thomas JW Penchansky R. Relating satisfaction with access to utilization of services. Med Care. (1984) 22(6):553–68. 10.1097/00005650-198406000-00006 6738145

[8] Wilson CR Rourke J Oandasan IF Bosco C. Progress made on access to rural health care in Canada. Can Fam Physician. (2020) 66(1):31–6. 10.4103/CJRM.CJRM_84_19 31854338

[9] Browne A. Issues Affecting Access to Health Services in Northern, Rural and Remote Regions of Canada. Prince-George, Canada: University of Northern British Columbia (2010).

[10] Colonna C. Rural and Remote Mental Health in Canada-Evidence Brief on Best and Promising. Ottawa, Canada: Mental Health Commission of Canada (2020).

[11] O'Connor A Wellenius G. Rural–urban disparities in the prevalence of diabetes and coronary heart disease. Public Health. (2012) 126(10):813–20. 10.1016/j.puhe.2012.05.029 22922043

[12] World Health Organization. Social determinants of health (2022). Available online at: https://www.who.int/health-topics/social-determinants-of-health#tab=tab_1 (accessed November 9, 2022).

[13] Leimbigler B Li EPH Rush KL Seaton CL. Social, political, commercial, and corporate determinants of rural health equity in Canada: an integrated framework. Can J Public Health. (2022) 113(5):749–54. 10.17269/s41997-022-00630-y 35437699 PMC9014974

[14] Reeve C Johnston K Young L. Health profession education in remote or geographically isolated settings: a scoping review. J Med Educ Curric Dev. (2020) 7:1–12. 10.1177/2382120520943595 PMC7378721 32754648

[15] Warren J Smalley KB. Rural Public Health: Best Practices and Preventive Models. New-York, NY: Springer Publishing Company (2014).

[16] Eberhardt MS Ingram DD Makuc DM. Urban and Rural Health Chartbook. Health, United States, 2001. Hyattsville, Maryland: National Center for Health Statistics (2001).

[17] AIHW. Rural, Regional and Remote Health: A Study on Mortality. Canberra, Australia: Australian Institute of Health and Welfare (2003).

[18] Mitura V Bollman RD. Health of rural Canadians: a rural-urban comparison of health indicators. Rural Small Town Canada Anal Bull. (2003) 4(6):1–23. Available online at: http://www.statcan.gc.ca/pub/21-006-x/21-006-x2002006-eng.pdf (accessed July 31, 2023).

[19] Hoffman PK Meier BP Council JR. A comparison of chronic pain between an urban and rural population. J Community Health Nurs. (2002) 19(4):213–24. 10.1207/S15327655JCHN1904_02 12494742

[20] Bath B Trask C McCrosky J Lawson J. A biopsychosocial profile of adult Canadians with and without chronic back disorders: a population-based analysis of the 2009–2010 Canadian community health surveys. BioMed Res Int. (2014) 2014:919621. 10.1155/2014/919621 24971357 PMC4058275

[21] Zimmer Z Fraser K Grol-Prokopczyk H Zajacova A. A global study of pain prevalence across 52 countries: examining the role of country-level contextual factors. Pain. (2022) 163(9):1740–50. 10.1097/j.pain.0000000000002557 35027516 PMC9198107

[22] Dahlhamer J Lucas J Zelaya C Nahin R Mackey S DeBar L Prevalence of chronic pain and high-impact chronic pain among adults—united States, 2016. MMWR Morb Mortal Wkly Rep. (2018) 67(36):1001–6. 10.15585/mmwr.mm6736a2 30212442 PMC6146950

[23] Yin Z Li S Ortega C Bobadilla R Winkler PL Hernández AE Impacts on patient-centered outcomes of a chronic pain self-management program in a rural community: a feasibility study. Geriatr Nurs (Minneap). (2021) 42(5):1198–203. 10.1016/j.gerinurse.2021.06.026 34425422

[24] Andrew R Derry S Taylor RS Straube S Phillips CJ. The costs and consequences of adequately managed chronic non-cancer pain and chronic neuropathic pain. Pain Pract. (2014) 14(1):79–94. 10.1111/papr.12050 23464879

[25] Gatchel RJ Peng YB Peters ML Fuchs PN Turk DC. The biopsychosocial approach to chronic pain: scientific advances and future directions. Psychol Bull. (2007) 133(4):581–624. 10.1037/0033-2909.133.4.581 17592957

[26] Hylands-White N Duarte RV Raphael JH. An overview of treatment approaches for chronic pain management. Rheumatol Int. (2017) 37(1):29–42. 10.1007/s00296-016-3481-8 27107994

[27] Canadian Pain Task Force. An Action Plan for Pain in Canada. Ottawa, Canada: Health Canada (2021).

[28] Eaton LH Langford DJ Meins AR Rue T Tauben DJ Doorenbos AZ. Use of self-management interventions for chronic pain management: a comparison between rural and nonrural residents. Pain Manag Nurs. (2018) 19(1):8–13. 10.1016/j.pmn.2017.09.004 29153296 PMC5807105

[29] Vorenkamp KE Kochat S Breckner F Dimon C. Challenges in utilizing telehealth for chronic pain. Curr Pain Headache Rep. (2022) 26(8):617–22. 10.1007/s11916-022-01067-1 35751799 PMC9244466

[30] Lacasse A Gagnon V Nguena Nguefack HL Gosselin M Pagé MG Blais L Chronic pain patients’ willingness to share personal identifiers on the web for the linkage of medico-administrative claims and patient-reported data: the chronic pain treatment cohort. Pharmacoepidemiol Drug Saf. (2021) 30(8):1012–26. 10.1002/pds.5255 33901339 PMC8360172

[31] Treede RD Rief W Barke A Aziz Q Bennett MI Benoliel R Chronic pain as a symptom or a disease: the IASP classification of chronic pain for the international classification of diseases (ICD-11). PAIN. (2019) 160(1):19–27. 10.1097/j.pain.0000000000001384 30586067

[32] CPN. (2017). *Chronic Pain Network Annual Report 2016/2017*. Hamilton (ON).

[33] Statistique Canada. Croissance Démographique Dans Les Régions Rurales du Canada, 2016 à 2021. Ottawa, Canada: Gouvernement du Canada (2022).

[34] Turk DC Dworkin RH Allen RR Bellamy N Brandenburg N Carr DB Core outcome domains for chronic pain clinical trials: IMMPACT recommendations. Pain. (2003) 106(3):337–45. 10.1016/j.pain.2003.08.001 14659516

[35] Dworkin RH Turk DC Farrar JT Haythornthwaite JA Jensen MP Katz NP Core outcome measures for chronic pain clinical trials: IMMPACT recommendations. Pain. (2005) 113(1-2):9–19. 10.1016/j.pain.2004.09.012 15621359

[36] Lacasse A Roy JS Parent AJ Noushi N Odenigbo C Pagé G The Canadian minimum dataset for chronic low back pain research: a cross-cultural adaptation of the national institutes of health task force research standards. CMAJ Open. (2017) 5(1):E237–e48. 10.9778/cmajo.20160117 28401140 PMC5378521

[37] Choinière M Ware MA Pagé MG Lacasse A Lanctôt H Beaudet N Development and implementation of a registry of patients attending multidisciplinary pain treatment clinics: the Quebec pain registry. Pain Res Manag. (2017) 2017:1–16. 10.1155/2017/8123812 PMC5322414 28280406

[38] CADTH. Chronic Pain Management: Non-Pharmacologic Treatments. Research Gaps: Improving the Evidence. (2018). Available online at: https://www.cadth.ca/sites/default/files/pdf/mindtheresearchgaps_chronic_pain_management_e.pdf (accessed July 31, 2023).

[39] Campbell F Hudspith M Anderson M Choiniere M El-Gabalawy H Laliberte J (2019). *Chronic Pain in Canada: Laying a foundation for action. A report by the Canadian Pain Task Force*.

[40] Masnoon N Shakib S Kalisch-Ellett L Caughey GE. What is polypharmacy? A systematic review of definitions. BMC Geriatr. (2017) 17(1):230. 10.1186/s12877-017-0621-2 29017448 PMC5635569

[41] Bouhassira D Attal N Alchaar H Boureau F Brochet B Bruxelle J Comparison of pain syndromes associated with nervous or somatic lesions and development of a new neuropathic pain diagnostic questionnaire (DN4). Pain. (2005) 114(1-2):29–36. 10.1016/j.pain.2004.12.010 15733628

[42] Sullivan MJ Bishop SR Pivik J. The pain catastrophizing scale: development and validation. Psychol Assess. (1995) 7(4):524. 10.1037/1040-3590.7.4.524

[43] Deyo RA Dworkin SF Amtmann D Andersson G Borenstein D Carragee E Report of the national institutes of health task force on research standards for chronic low back pain. J Manipulative Physiol Ther. (2014) 37(7):449–67. 10.1016/j.jmpt.2014.07.006 25127996

[44] Bruyère O Demoulin M Brereton C Humblet F Flynn D Hill JC Translation validation of a new back pain screening questionnaire (the STarT back screening tool) in French. Arch Public Health. (2012) 70(1):12. 10.1186/0778-7367-70-12 22958224 PMC3436683

[45] Cleeland CS. The Brief Pain Inventory User Guide. Houston, TX: The University of Texas MD Anderson Cancer Center (2009). p. 1–11.

[46] Ware JE Kosinski M Turner-Bowker DM Gandek B. User’s Manual for the SF-12v2 Health Survey. Lincoln, RI: QualityMetric (2009).

[47] Kroenke K Spitzer RL Williams JB Löwe B. An ultra-brief screening scale for anxiety and depression: the PHQ-4. Psychosomatics. (2009) 50(6):613–21. 10.1176/appi.psy.50.6.613 19996233

[48] Reid S Day C White N Harrison C Haber P Bayram C. Opioid prescribing in general practice: an Australian cross-sectional survey. BMC Primary Care. (2022) 23(1):171. 10.1186/s12875-022-01783-y 35804306 PMC9264661

[49] Larance B Campbell G Peacock A Nielsen S Bruno R Hall W Pain, alcohol use disorders and risky patterns of drinking among people with chronic non-cancer pain receiving long-term opioid therapy. Drug Alcohol Depend. (2016) 162:79–87. 10.1016/j.drugalcdep.2016.02.048 27049582

[50] Choinière M Peng P Gilron I Buckley N Williamson O Janelle-Montcalm A Accessing care in multidisciplinary pain treatment facilities continues to be a challenge in Canada. Reg Anesth Pain Med. (2020) 45(12):943–8. 10.1136/rapm-2020-101935 33024007

[51] Coughlin LN Bonar EE Bohnert KM Jannausch M Walton MA Blow FC Changes in urban and rural cigarette smoking and cannabis use from 2007 to 2017 in adults in the United States. Drug Alcohol Depend. (2019) 205:107699. 10.1016/j.drugalcdep.2019.107699 31707265 PMC6951810

[52] Schell C Godinho A Cunningham JA. Examining the influence of rurality on frequency of cannabis use and severity of consequences as moderated by age and gender. Addict Behav. (2022) 133:107385. 10.1016/j.addbeh.2022.107385 35687936

[53] Coughlin LN Walton MA McCormick R Blow FC. Prevalence and severity of alcohol and Cannabis use across the urban-rural continuum in the Michigan national guard. J Rural Health. (2020) 36(2):234–9. 10.1111/jrh.12412 31840327 PMC7102927

[54] Azagba S Shan L Latham K. Rural-urban differences in cannabis detected in fatally injured drivers in the United States. Prev Med. (2020) 132:105975. 10.1016/j.ypmed.2019.105975 31899254

[55] Ambrose CA Cowan BW Rosenman RE. Geographical acces to recreational marijuana. Contemp Econ Policy. (2021) 39(4):778–807. 10.1111/coep.12518 34712040 PMC8547494

[56] Institut de la statistique du Québec. Enquête québécoise sur le cannabis 2021: La consommation de cannabis et les perceptions des Québécois. Portrait et évolution de 2018 à 2021. Quebec, Canada Gouvernement du Québec (2022).

[57] Radio-Canada. La succursale de la SQDC ouvrira le 20 mai à Val-d’Or (2020). Available online at: https://ici.radio-canada.ca/ohdio/premiere/emissions/region-zero-8/segments/entrevue/170344/ouverture-succursale-sqdc-valdor (accessed July 31, 2023).

[58] Cantin A. Première succursale de la SQDC sur la côte-nord. J Québec. (2020). Available online at: https://www.journaldequebec.com/2020/08/25/premiere-succursale-de-la-sqdc-sur-la-cote-nord (accessed July 31, 2023).

[59] LP Canadienne. La SQDC affiche un profit en hausse de 13,3% (2022). Available online at: https://www.lapresse.ca/affaires/entreprises/2022-02-17/troisieme-trimestre/la-sqdc-affiche-un-profit-en-hausse-de-13-3.php (accessed July 31, 2023).

[60] IASP. International association for the study of pain presidential task force on Cannabis and cannabinoid analgesia position statement. Pain. (2021) 162:S1–2. 10.1097/j.pain.0000000000002037 33729207

[61] Clay FJ Watson WL Newstead SV McClure RJ. A systematic review of early prognostic factors for persisting pain following acute orthopedic trauma. Pain Res Manag. (2012) 17(1):35–44. 10.1155/2012/935194 22518366 PMC3299034

[62] Kyriacou DN Lewis RJ. Confounding by indication in clinical research. JAMA. (2016) 316(17):1818–9. 10.1001/jama.2016.16435 27802529

[63] Pong RW DesMeules M Lagacé C. Rural–urban disparities in health: how does Canada fare and how does Canada compare with Australia? Aust J Rural Health. (2009) 17(1):58–64. 10.1111/j.1440-1584.2008.01039.x 19161503

[64] IALA. Plan Stratégique 2021-2026 de l'IALA des IRSC. Ottawa, Canada: Instituts de recherche en santé du Canada (2021).

