# Supplementary material for: Where we live matters: a comparison of chronic pain treatment between remote and non-remote regions of Quebec, Canada
Source: Front Pain Res (Lausanne). 2024 Feb 26;5:1291101. doi: 10.3389/fpain.2024.1291101 (PMC10925759; doi:10.3389/fpain.2024.1291101)
Supplement: Supplementary file 1 [file Datasheet1.pdf]

SUPPLEMENTARY MATERIAL – Complete results from the multivariable analyses.

	Use of prescribed pain medication	Use of over-the-counter pain medications	Use of non-pharmacological treatments	Use of a multimodal approach	Access to a trusted health care professional for pain management	Excessive polypharmacy (use of ≥10 medications)	Use of cannabis for pain management
Hosmer-Lemeshow test, chi-square (*p*)	5.843 (0.665)	5.775 (0.672)	11.240 (0.188)	6.419 (0.600)	6.831 (0.555)	8.193 (0.415)	8.329 (0.402)
OR (95% CI)							
Main independent variable							
Living in a remote region (yes vs no)	**0.60 (0.41-0.87)**	1.02 (0.74-1.41)	0.89 (0.56-1.40)	0.76 (0.51-1.14)	0.82 (0.65-1.20)	0.94 (0.63-1.40)	**0.60 (0.38-0.94)**
Sociodemographic profile							
Age (years)	0.99 (0.97-1.00)	**1.02 (0.99-1.03)**	**0.98 (0.96-0.99)**	0.99 (0.97-1.00)	1.00 (0.99-1.02)	**1.02 (1.01-1.03)**	**0.97 (0.96-0.99)**
Self-identified gender (women vs other)	1.00 (0.62-1.62)	**1.59 (1.11-2.29)**	1.20 (0.73-1.95)	**1.69 (1.09-2.62)**	1.02 (0.66-1.58)	0.93 (0.60-1.44)	0.90 (0.55-1.45)
Aboriginal identity (yes versus no)	1.29 (0.32-5.13)	1.02 (0.38-2.71)	0.38 (0.12-1.20)	0.58 (0.18-1.81)	1.17 (0.36-3.80)	0.77 (0.22-2.68)	0.74 (0.18-2.99)
Country of birth (Canada vs other)	1.10 (0.47-2.60)	0.94 (0.47-1.87)	0.66 (0.24-1.86)	1.22 (0.53-2.78)	1.70 (0.81-3.59)	2.63 (0.99-7.03)	0.62 (0.28-1.38)
Employed full or part-time (yes vs no)	1.00 (0.68-1.46)	1.35 (0.99-1.83)	0.81 (0.52-1.27)	0.72 (0.48-1.07)	0.86 (0.59-1.24)	**0.56 (0.39-0.82)**	0.87 (0.59-1.30)
Postsecondary education (yes vs no)	**1.86 (1.20-2.90)**	1.17 (0.83-1.65)	**2.73 (1.80-4.15)**	**2.56 (1.71-3.83)**	0.90 (0.59-1.37)	0.88 (0.60-1.29)	1.39 (0.88-2.19)
Chronic pain characteristics and interference							
Localization (yes vs no)							
- Neck	1.32 (0.86-2.03)	1.18 (0.84-1.66)	**1.70 (1.02-2.84)**	**1.76 (1.12-2.77)**	1.18 (0.79-1.78)	1.30 (0.87-1.94)	0.64 (0.41-1.01)
- Shoulders	0.90 (0.86-2.03)	1.24 (0.88-1.74)	0.69 (0.42-1.16)	**0.58 (0.36-0.91)**	1.17 (0.78-1.75)	0.87 (0.58-1.29)	1.29 (0.83-2.01)
- Back	1.06 (0.71-1.58)	1.25 (0.90-1.75)	1.28 (0.78-2.09)	1.35 (0.88-2.07)	0.71 (0.47-1.06)	0.84 (0.55-1.28)	0.70 (0.45-1.09)
- Hips	0.77 (0.52-1.16)	**0.69 (0.50-0.95)**	1.17 (0.76-1.89)	1.13 (0.73-1.75)	1.07 (0.74-1.57)	1.20 (0.82-1.76)	1.24 (0.82-1.90)
- Legs	1.09 (0.72-1.65)	1.02 (0.73-1.41)	0.68 (0.42-1.80)	0.82 (0.53-1.25)	1.08 (0.74-1.59)	1.24 (0.85-1.80)	1.30 (0.86-1.96)
Multisite (yes vs no)	0.89 (0.51-1.53)	1.20 (0.77-1.88)	**1.93 (1.04-3.60)**	1.48 (0.84-2.60)	0.54 (0.30-0.99)	1.01 (0.54-1.89)	1.43 (0.72-2.81)
Frequency (continuous vs intermittent)	**1.85 (1.17-2.92)**	1.00 (0.65-1.53)	1.07 (0.55-2.08)	1.22 (0.72-2.09)	1.19 (0.72-1.97)	1.31 (0.70-2.44)	1.84 (0.94-3.60)
Duration ≥ 10 years (yes vs no)	1.35 (0.95-1.90)	0.78 (0.59-1.01)	1.43 (0.97-2.12)	1.24 (0.87-1.77)	0.87 (0.63-1.20)	0.90 (0.65-1.23)	1.21 (0.85-1.72)
Intensity on average in the past 7 days (0-10 NRS)	1.05 (0.94-1.18)	**0.91 (0.83-0.99)**	0.89 (0.79-1.02)	0.92 (0.82-1.03)	1.07 (0.96-1.19)	1.17 (1.05-1.31)	0.91 (0.81-1.02)
Neuropathic component according to the DN4 (yes vs no)	1.23 (0.86-1.77)	0.95 (0.72-1.26)	1.03 (0.69-1.54)	1.05 (0.73-1.53)	0.97 (0.69-1.36)	1.02 (0.73-1.41)	1.24 (0.86-1.79)
Interference (BPI score)	1.03 (0.91-1.15)	0.99 (0.90-1.08)	1.00 (0.88-1.15)	1.02 (0.90-1.15)	1.00 (0.89-1.19)	1.00 (0.90-1.13)	1.11 (0.98-1.26)
Tendency to pain catastrophizing (yes vs no)	**1.72 (1.15-2.55)**	1.02 (0.75-1.40)	**0.61 (0.38-0.99)**	0.97 (0.64-1.46)	0.74 (0.50-1.09)	**0.68 (0.47-0.99)**	1.17 (0.78-1.74)

Pain treatment							
Use of prescribed pain medications (yes vs no)	-	0.97 (0.68-1.38)	**0.46 (0.26-0.81)**	-	**2.74 (1.87-4.01)**	**3.82 (2.14-6.80)**	**2.69 (1.56-4.63)**
Use of over-the-counter pain medications (yes vs no)	0.90 (0.62-1.30)	-	**1.91 (1.31-2.79)**	**5.57 (3.93-7.90)**	1.12 (0.80-1.57)	1.10 (0.79-1.54)	**0.62 (0.43-0.89)**
Use of non-pharmacological treatments (yes vs no)	**0.50 (0.29-0.88)**	**1.90 (1.31-2.76)**	**-**	-	1.42 (0.91-2.23)	1.50 (0.95-2.35)	**2.01 (1.16-3.49)**
Access to a trusted health care professional for pain management (yes vs no)	**2.89 (1.96-4.26)**	1.14 (0.81-1.59)	**1.56 (0.99-2.44)**	**1.87 (1.26-2.78)**	-	1.42 (0.93-2.18)	1.30 (0.83-2.04)
Excessive polypharmacy (yes vs no)	**3.60 (2.02-6.42)**	1.09 (0.79-1.50)	1.48 (0.94-2.33)	**1.72 (1.10-2.68)**	1.41 (0.93-2.14)	-	1.00 (0.67-1.49)
Cannabis for pain (yes vs no)	**2.63 (1.53-4.53)**	**0.66 (0.47-0.93)**	**2.04 (1.17-3.54)**	**2.72 (1.62-4.56)**	1.24 (0.80-1.93)	1.05 (0.70-1.57)	-
Pain relief brought by treatments (%)	**1.02 (1.01-1.03)**	**0.99 (0.99-0.99)**	1.00 (0.99-1.01)	1.00 (0.99-1.02)	**1.02 (1.01-1.02)**	**1.01 (1.01-1.02)**	**0.98 (0.97-0.99)**
Health profile and lifestyle							
Psychological distress PHQ4 score vs none/scores 0-2)							
- Mild (3-5)	1.12 (0.73-1.73)	1.27 (0.89-1.82)	1.43 (0.83-2.47)	1.36 (0.84-2.18)	**0.55 (0.35-0.87)**	0.82 (0.53-1.28)	1.48 (0.91-2.40)
- Moderate (6-8)	0.86 (0.52-1.44)	1.04 (0.70-1.57)	1.15 (0.64-2.09)	1.00 (0.59-1.70)	0.71 (0.42-1.21)	0.89 (0.55-1.45)	1.13 (0.66-1.96)
- Severe (9-12)	1.58 (0.82-3.03)	1.31 (0.82-2.10)	1.08 (0.56-2.06)	1.12 (0.61-2.04)	**0.49 (0.27-0.86)**	0.86 (0.50-1.45)	0.74 (0.39-1.39)
Physical functioning (0-100 SF-12 score)	**0.97 (0.95-0.99)**	0.99 (0.97-1.01)	**0.98 (0.95-0.99)**	**0.97 (0.95-0.99)**	1.01 (0.99-1.03)	**0.96 (0.94-0.98)**	1.00 (0.98-1.02)
Have consumed alcohol or used drugs more than intended in the past year (versus never)							
- Rarely	0.81 (0.54-1.21)	1.14 (0.82-1.58)	1.14 (0.70-1.89)	1.31 (0.84-2.02)	**1.64 (1.10-2.47)**	1.03 (0.70-1.53)	1.28 (0.84-1.93)
- Sometimes	1.25 (0.71-2.18)	**1.72 (1.10-2.70)**	1.00 (0.54-1.86)	1.05 (0.60-1.84)	1.15 (0.69-1.93)	0.92 (0.55-1.53)	1.73 (1.03-2.92)
- Often	1.38 (0.55-3.44)	1.02 (0.55-1.90)	0.56 (0.25-1.25)	0.75 (0.35-1.61)	1.59 (0.72-3.49)	1.35 (0.68-2.69)	1.46 (0.66-3.24)
Perceived general health (0–100 SF-12 score)	0.99 (0.98-1.01)	0.99 (0.98-1.01)	**1.05 (1.03-1.07)**	**1.04 (1.02-1.05)**	1.01 (0.99-1.02)	**0.95 (0.94-0.96)**	0.99 (0.98-1.01)
Cigarette smoking (vs never smoked)							
- Current smoker	0.81 (0.47-1.39)	0.94 (0.63-1.40)	0.66 (0.40-1.12)	**0.54 (0.33-0.88)**	**0.62 (0.39-0.99)**	0.87 (0.55-1.38)	**2.32 (1.40-3.86)**
- Former smoker	1.17 (0.81-1.70)	1.02 (0.76-1.37)	1.09 (0.71-1.66)	1.00 (0.68-1.48)	0.95 (0.66-1.35)	1.16 (0.82-1.62)	**1.89 (1.29-2.77)**
Cannabis for other illnesses (yes vs no)	0.63 (0.30-1.33)	1.20 (0.71-2.01)	0.93 (0.42-2.08)	0.82 (0.40-1.69)	1.25 (0.65-2.41)	1.32 (0.74-2.36)	**-**
Cannabis for recreational purpose (yes vs no)	**0.51 (0.28-0.94)**	1.02 (0.65-1.61)	0.71 (0.35-1.45)	**0.50 (0.27-0.91)**	0.65 (0.38-1.11)	1.03 (0.59-1.81)	**-**

Table footnotes: BPI = Brief Pain Inventory; CI = Confidence interval; DN4 = Questionnaire Douleur Neuropathique 4 questions; NRS = Numerical Rating Scale; OR = Odds ratio; PHQ-4 = 4 item Patient Health Questionnaire; SF-12 = 12-Item Short Form Survey v2; Statistically significant associations (p<.05) are reported in bold.
